# A single-cell atlas of in vitro multiculture systems uncovers the in vivo lineage trajectory and cell state in the human lung

**DOI:** 10.1038/s12276-023-01076-z

**Published:** 2023-08-15

**Authors:** Woochan Lee, Seyoon Lee, Jung-Ki Yoon, Dakyung Lee, Yuri Kim, Yeon Bi Han, Rokhyun Kim, Sungji Moon, Young Jun Park, Kyunghyuk Park, Bukyoung Cha, Jaeyong Choi, Juhyun Kim, Na-young Ha, Kwhanmien Kim, Sukki Cho, Nam-Hyuk Cho, Tushar J. Desai, Jin-Haeng Chung, Joo-Hyeon Lee, Jong-Il Kim

**Affiliations:** 1 Department of Biomedical Sciences, Seoul National University College of Medicine, Seoul, Korea; 2 Division of Pulmonary, Allergy and Critical Care, Department of Medicine, Stanford University School of Medicine, Stanford, CA USA; 3 Institute of Endemic Diseases, Medical Research Center, Seoul National University, Seoul, Korea; 4 Department of Pathology and Translational Medicine, Seoul National University Bundang Hospital, Seongnam, Korea; 5 Interdisciplinary Program in Cancer Biology, College of Medicine, Seoul National University, Seoul, Korea; 6 Department of Translational Medicine, Seoul National University College of Medicine, Seoul, Korea; 7 Genomic Medicine Institute (GMI), Medical Research Center, Seoul National University, Seoul, Korea; 8 Department of Thoracic and Cardiovascular Surgery, Seoul National University Bundang Hospital, Seongnam, Korea; 9 Department of Microbiology and Immunology, Seoul National University College of Medicine, Seoul, Korea; 10 Wellcome–MRC Cambridge Stem Cell Institute, Jeffrey Cheah Biomedical Centre, University of Cambridge, Cambridge, UK; 11 Department of Physiology, Development and Neuroscience, University of Cambridge, Cambridge, UK; 12 Department of Biochemistry and Molecular Biology, Seoul National University College of Medicine, Seoul, Korea; 13 Cancer Research Institute, Seoul National University, Seoul, Korea

**Keywords:** Genetic databases, Transcriptomics, Stem-cell differentiation

## Abstract

We present an in-depth single-cell atlas of in vitro multiculture systems on human primary airway epithelium derived from normal and diseased lungs of 27 individual donors. Our large-scale single-cell profiling identified new cell states and differentiation trajectories of rare airway epithelial cell types in human distal lungs. By integrating single-cell datasets of human lung tissues, we discovered immune-primed subsets enriched in lungs and organoids derived from patients with chronic respiratory disease. To demonstrate the full potential of our platform, we further illustrate transcriptomic responses to various respiratory virus infections in vitro airway models. Our work constitutes a single-cell roadmap for the cellular and molecular characteristics of human primary lung cells in vitro and their relevance to human tissues in vivo.

## Organ models: Checking a match with human lung tissue

A new atlas of single cell profiles from human lungs provides a powerful platform for comparing immune responses in vitro and in vivo. The COVID-19 pandemic highlighted the usefulness of growing mini human organ cultures, called organoids, to understand the pathogenesis of diseases, especially in the epithelial cells that line the lungs. However, it is vital to confirm that these model systems accurately replicate the gene expression of real tissues. Jong-Il Kim at Seoul National University and co-workers established a biobank of 83 organoid lines derived from 27 healthy and diseased donor lungs, and verified that their transcriptomes—single cell mRNA expression profiles – were similar to those of real human lung tissues. This enabled them to map the differentiation of resident stem cells into rare airway types, and discover distinct subsets of cells primed for immunity.

## Introduction

Single-cell transcriptomics has increased knowledge of the cellular and molecular composition of human tissues, including the identification of previously unknown cell types and states, some of which arise specifically during human diseases^1–5^. However, due to the limited tools for growing and manipulating human cells in vitro, the functional roles for these populations have yet to be fully elucidated. Over the past decade, three-dimensional (3D) culture systems known as organoids have been developed to grow ‘mini human organs’ derived from stem cells that self-renew and differentiate into multiple cell lineages^6^. Given their ability to recapitulate key aspects of in vivo organs, organoid models opened up new avenues for studying human organ development. Significantly, the current coronavirus disease 2019 (COVID-19) pandemic caused by severe acute respiratory syndrome coronavirus 2 (SARS-CoV-2) highlighted the importance of these biological platforms to better understand the pathogenesis of human diseases^7–13^. Before expanding their use in clinical applications and translational research, it is critical to determine whether the gene expression programs and lineage relationships in vitro models faithfully replicate those of their respective tissues.

The respiratory epithelium is a primary site of environmental exposures, including viruses, which can lead to life-threatening respiratory failure. Human lung stem cell-derived in vitro culture systems have emerged as powerful tools for studying human lung development and modeling pulmonary diseases. The air-liquid interface (ALI) 2D culture system is well established and involves the culture of airway epithelial cells such that the basal surface of the cells is in contact with the medium while the apical surface is exposed to air^14–16^. In this system, basal cells expand and undergo proper differentiation into a pseudostratified monolayer barrier, consisting of ciliated and secretory cells, providing a robust in vitro airway model. The airway 3D organoids derived from basal or club cells can be maintained in the long term and generate differentiated cells, allowing for the study of cellular lineage hierarchy in the human airway epithelium^17–19^. Current COVID-19 research, in particular, has fueled the application of these platforms in the study of viral tropism and infection pathogenesis of SARS-CoV-2 in human airways^20–24^. However, the fidelity of these two model systems for the prospective modeling of infection has not been thoroughly validated.

Here, we establish biobanks of 83 human primary airway organoid lines and present a single-cell atlas derived from a subset of 27 individuals. We directly compared the transcriptomes of 2D and 3D human airway in vitro models against in vivo human lung tissues by incorporating public datasets from the Human Lung Cell Atlas^1^. Our deep single-cell profiling enabled us to map the differentiation trajectories of rare airway cell types emerging from basal cells over pseudotime. We also discovered distinct immune-primed subsets in vitro models derived from the lungs of patients with chronic respiratory diseases that are also enriched in vivo lungs. In addition, we demonstrate the utility of in vitro models and our organoid single-cell atlas by comparing cellular states and immune responses following various viral infections at the single-cell level. Our organoid single-cell atlas datasets constitute a valuable resource for interactive exploration by the research community online (OSCA: http://osca.snu.ac.kr).

## Methods

### Human tissues

For the establishment of human primary distal airway organoid cultures, human distal lung tissues were obtained from lung cancer patients undergoing lung resection surgery at Seoul National University Bundang Hospital (SNUH) who provided written informed consent with the approval of the ethical committee (IRB No. 2008-065-1148). Organoids were derived from the lung tissues farthest from tumor lesions without (normal lung tissues) or with a history of chronic respiratory diseases (diseased lung tissues) (Supplementary Table 1). Fresh lung tissues were stored in media (Advanced DMEM/F12 (Gibco), 1% HEPES (Gibco), 1% Glutamax (Gibco), and 0.125 µg/ml amphotericin B (Gibco)) at 4 °C for less than 12 h before processing.

### Isolation and culture of human primary distal airway organoids

Lung tissues were cut into < 5 mm pieces and washed in ice-cold PBS to remove residual mucus and blood cells. Before snap freezing for DNA extraction, 2-3 pieces of lung tissue were stored in RNAlater solution (Invitrogen) overnight at 4 °C. The remaining tissue pieces were dissociated into single cells using a gentleMACS Octo Dissociator with Heater (Miltenyi Biotec) according to the manufacturer’s protocol. The tissue suspensions were filtered through a 70 μm cell strainer (Miltenyi Biotec) and washed with 5 ml of Advanced DMEM/F12. Cells were centrifuged at 400 × *g* for 10 min at 4 °C, and the supernatant was aspirated. Cell pellets were resuspended in 1 ml of red blood cell lysis buffer (Miltenyi Biotec) for 10 min at room temperature (RT). The reaction was quenched by adding 9 ml of Advanced DMEM/F12 with 1% HEPES, 1% Glutamax, and 1% penicillin-streptomycin (Gibco) (hereafter ADF + ++), followed by centrifugation at 400 × *g* for 10 min at 4 °C. Cell pellets were then resuspended in growth factor-reduced Matrigel® (GFR-Matrigel; Corning) and plated as 40 μl droplets in a prewarmed 24-well tissue culture plate. Plates were incubated at 37 °C for 15 min, followed by submersion in 500 μl of prewarmed airway organoid medium (AO medium) with 10 µM Y-27632 (Tocris) for the first 3 days^17^. The medium was changed every 3 days. Established three-dimensional (3D) airway organoids (3D-AOs) were passaged at a 1:4 ratio every 1-2 weeks. For passaging, TrypLE Express (Gibco) was added to each well, and GFR-Matrigel was mechanically disrupted. Organoids were incubated at 37 °C for 5 min. The reaction was quenched by adding ice-cold ADF + ++, and cells were centrifuged at 400 × *g* for 5 min at 4 °C. Cells were resuspended in GFR-Matrigel and plated, as described above. For cytokine treatment, we added 1 ng/ml IL-13 (Biolegend), 10 ng/ml IL-1β (Biolegend) or 10 ng/ml IL-6 (R&D Systems) to 3D-AOs for 3 weeks.

### Air-liquid interface (ALI) culture of human primary distal airway organoids

Established 3D-AOs were dissociated into single cells as described above. Transparent 24-well transwell inserts (Corning) were precoated with 1% v/v GFR-Matrigel for at least 1 h at 37 °C. Dissociated 200 K airway cells were then resuspended in 200 μL of AO medium or PneumaCult^TM^-Ex Plus medium (STEMCELL Technologies) and seeded in each Transwell insert for 2D ALI cultures (ALI-Om or ALI-Pm, respectively). Then, 500 μL of medium was added to the lower compartment of the transwell plates. After 5–8 days, when monolayers reached 100% confluency, growth medium was removed from the inserts to induce differentiation for another 21 days. For ALI-Pm, PneumaCult^TM^-ALI Medium (STEMCELL Technologies) was used to induce differentiation.

### Single-cell RNA sequencing of in vitro human primary airway cultures

Established 3D-AOs between passages 3 and 10 from individual donors were used for sequencing. 3D-AOs were dissociated into single cells and plated to establish ALI-Om and ALI-Pm, which were used for sequencing after 3 weeks of differentiation. Dissociated cells were resuspended in 1 ml ADF + ++ and filtered through a 30 μm SmartStrainer (Miltenyi Biotec). Each sample was then analyzed using ReadyCount Green/Red Viability Stain (Invitrogen) to count the number of cells and measure cell viability with the automated cell counter Countess 3 FL (Thermo Fisher). Cell suspensions from in vitro cultures established from different donors were pooled to obtain a cell suspension mixture with equal proportions of each sample. The pooled cell suspension was centrifuged at 400 × g for 5 min at 4 °C and then resuspended in 0.04% bovine serum albumin (BSA) solution at an appropriate volume for microfluidic chip loading. Typically, 6 to 8 samples were loaded per lane of a 10x microfluidic chip device and demultiplexed based on single-nucleotide polymorphisms. For 10x multiome analysis, one sample was loaded on a Chromium Next GEM Chip J. All libraries were sequenced using a NovaSeq 6000 system (Illumina) in paired-end mode.

### Immunofluorescence staining of 2D ALI and 3D cultures

For immunofluorescence (IF) staining of 3D-AOs, Matrigel was dissolved in ice-cold Cell Recovery Solution (Corning) at 60 rpm for 30 to 60 min at 4 °C on a horizontal shaker (CRYSTE). The recovered organoids were fixed in 4% paraformaldehyde for 30 min at 4 °C. For IF staining of ALI-Oms, the insert was displaced from the plate and placed upside down on a clean petri dish. Using a surgical blade, the membrane with cells was detached from the insert and fixed in 4% paraformaldehyde for 30 min at 4 °C. The samples were then washed with 0.1% Triton X-100/PBS (PBS-T). They were then permeabilized in 0.2% Triton X‐100/PBS for 5 min and incubated in 5% donkey serum (Jackson ImmunoResearch)/PBS-T blocking solution for 2 h at RT. Primary antibodies were incubated overnight at 4 °C at the indicated dilutions: chicken anti-CK5 (1:500, Biolegend, 905904), mouse anti-TP63 (1:100, Thermo Fisher, MA1-21871), rabbit anti-MUC5AC (1:200, Cell Signaling Technology, 61193), rat anti-SCGB1A1 (1:200, R&D Systems, MAB4218), rabbit anti-acetyl-α-tubulin (1:200, Cell Signaling Technology, 5335), mouse anti-SARS-CoV NP (1:150, Sino Biological, 40143-MM05), mouse anti-influenza A NP (1:100, Meridian, C87050M), and rabbit anti‐SARS‐CoV NP (1:1000, Sino Biological, 40143‐T62). The samples were incubated overnight with Alexa Fluor-coupled secondary antibodies (1:500, Invitrogen) at 4 °C. After antibody staining, nuclei were stained with DAPI (1:1000, Sigma), and sections were embedded in Vectashield Plus antifade mounting medium (H-1900, Vector Laboratories). Fluorescence images were acquired using either Leica SP8 or LSM700 confocal microscopes. LAS X (Leica) or ZEN software (Zeiss) was used for processing fluorescent images.

### Quantitative PCR

Total RNA was extracted from 3D-AOs and ALI-Oms using the RNeasy Mini Kit (Qiagen). Complementary DNA (cDNA) was synthesized from extracted RNA using a 1^st^ strand cDNA synthesis kit (Takara) according to the manufacturer’s protocols. Primers for *ACE2*, *TMPRSS2*, and *GAPDH* are listed in Supplementary Table 3. Quantitative PCR (q-PCR) was performed with a Viia7 real-time PCR system (Thermo Fisher) using TB Green Premix Ex Taq II (Takara) according to the manufacturer’s instructions. Relative expression (2^-ddCt^) was normalized to the Ct of GAPDH and dCt of 3D-AOs.

### Virus preparation

SARS-CoV-2 alpha and delta strains were obtained from the Korean National Culture Collection for Pathogens (NCCP nos. 43326 and 43390, respectively). Viruses included in the beta or omicron lineage were isolated from nasopharyngeal swabs taken from COVID-19 patients (GenBank accession nos. OP349649.1 and 349650.1, respectively). Wild-type MERS-CoV (GenBank accession no. KT029139.1) was provided by the Korean Centers for Disease Control (KCDC). MERS-CoV and SARS-CoV-2 were propagated on VeroE6 cells (CRL-1586, ATCC) in DMEM (Welgene) supplemented with 2% fetal bovine serum (FBS, Gibco) and 100 IU/ml penicillin‒streptomycin at 37 °C in a humidified CO_2_ incubator for 3 days and titrated under overlay medium containing 0.8% methylcellulose (Sigma) and 2% FBS in DMEM. Influenza A (VR-95™, ATCC) was propagated in embryonated chicken eggs and titrated in MDCK cells (CCL-34™, ATCC) by plaque assay. The culture supernatant was cleared by centrifugation and stored in aliquots at −80 °C until use.

### Viral infection

All work with infectious viruses was performed in a Class II Biosafety Cabinet under BSL‐3 (MERS-CoV, SARS-CoV-2) or BSL-2 (influenza A) conditions at Seoul National University. For all virus (SARS-CoV-2, MERS-CoV, and influenza A) infections of 2D differentiated cultures, samples were washed twice with 200 µL ADF + ++ before infection from the apical side of the ALI culture at an MOI of 1.0. The number of cells in each culture was calculated by counting the cells from 1 equally cultured well. The cultures were incubated at 37 °C and 5% CO_2_ for 2 h before washing three times in 200 μL ADF + ++. The samples were then cultured under normal culture conditions with AO medium in the lower compartment for 3 days before the generation of the scRNA-seq library, IF staining, or transmission electron microscopy (TEM).

### Transmission electron microscopy

To observe SARS-CoV-2 virus particles using TEM, infected ALI-Oms were fixed with a mixture of 2.5% glutaraldehyde in 0.1 M phosphate buffer (pH 7.2) and 2% paraformaldehyde in 0.1 M phosphate buffer (pH 7.2) for 3 h^25–27^. The intact polyester membranes with fixed cell layers were carefully removed from the transwell inserts. The intact membrane disks with cell layers were subsequently fixed for 1.5 h at RT with 2% osmium tetroxide in 0.1 M phosphate buffer. The samples were dehydrated in graded ethanol and then infiltrated with propylene oxide and EPON epoxy resin. The samples were then embedded in EMbed-812 resin (EMS) and polymerized at 80 °C overnight. The samples were cut on an ultramicrotome (RMC MT-XL) at 65 nm. Ultrathin sections were stained with saturated 4% uranyl acetate and 4% lead citrate. TEM imaging and analysis were carried out using a transmission electron microscope (JEM-1400) at 80 kV in the Department of Research & Experiment at Seoul National University Hospital.

### K-chip genotype array

DNA was separated and extracted from frozen tissue or organoids. DNA genotyping was performed using Korea Biobank Array V1.1 (ThermoFisher)^28^. The Korea Biobank Array Project analytic protocol was used to preprocess the data, and minor allele variations with low minor allele frequencies were included. Variants in linkage disequilibrium were excluded. Plink v1.0.9 was used to perform genotype principal component analysis, and imputation was performed using the East Asian-specific WGS imputation reference panel (Northeast Asian Reference Database V2, unpublished)^29,30^. Beagle5 and Minimac4 were used to perform phasing and imputation, respectively^31,32^. Variants were chosen using Bcftools and Plink 2.00a3 according to the following criteria: (i) imputation R2 higher than 0.8, (ii) Hardy–Weinberg equilibrium *p* value higher than 1e-6, (iii) variant call rate higher than 0.9, and (iv) minor allele frequency higher than 0.05^33^.

### Computational methods

#### Constructing an organoid single-cell atlas (OSCA) by analysis of scRNA-seq data

We used Cell Ranger (v6.0.1)^34^ to align sequenced reads to the GRCh38 human reference genome. For demultiplex individuals, we used souporcell (v2.0)^35^ with donors’ genotype array data from the K-chip. All samples underwent ambient RNA removal using cellbender (v0.2.1)^36^ to remove background noise expression and doublets. Only cells that were assigned a single donor, passed the doublet filter, expressed more than 200 genes, and did not express more than 10% of mitochondrial genes were used for further analysis. SCTransformation in the Seurat package (v4.0.3)^37^ regressing out mitochondrial gene percentage (percent.mt) was performed for normalization. We performed canonical correlation analysis (CCA) with up to 50 PCs as the integration method to correct the batch effect between each sequencing library. RunPCA, RunUMAP, and FindNeighbors followed by FindClusters with the Louvain algorithm in the Seurat package were used to visualize the UMAP plot and cluster cells. Analysis of sequencing data was mostly carried out using the computing server at the Genomic Medicine Institute Research Service Center.

### Cell cycle scoring

To compare the proportions of each cell cycling phase between culture methods, we used CellCycleScoring in the Seurat package^37^. “MCM”, “PCN”, “TYM”, “FEN”, “MCM”, “MCM”, “RRM”, “UN”, “GINS”, “MCM”, “CDCA”, “DT”, “PRIM”, “UHRF”, “MLF1I”, “HELL”, “RFC”, “RPA”, “NAS“, “RAD51AP”, “GMN”, “WDR7”, “SLB”, “CCNE”, “UBR”, “POLD”, “MSH”, “ATAD”, “RAD5”, “RRM”, “CDC4”, “CDC”, “EXO”, “TIPI”, “DSCC”, “BL”, “CASP8AP”, “USP”, “CLSP”, “POLA”, “CHAF1”, “BRIP”, and “E2F” were used as marker genes of S phase, and “HMGB”, “CDK”, “NUSAP”, “UBE2”, “BIRC”, “TPX”, “TOP2”, “NDC8”, “CKS”, “NUF”, “CKS1“, “MKI6”, “TMP”, “CENP”, “TACC”, “FAM64”, “SMC”, “CCNB”, “CKAP2”, “CKAP”, “AURK”, “BUB”, “KIF1”, “ANP32”, “GTSE”, “KIF20”, “HJUR”, “CDCA”, “HN”, “CDC2”, “TT”, “CDC25”, “KIF2”, “RANGAP”, “NCAPD”, “DLGAP”, “CDCA”, “CDCA”, “ECT”, “KIF2”, “HMM”, “AURK”, “PSRC”, “ANL”, “LB”, “CKAP”, “CENP”, “CTC”, “NEK”, “G2E”, “GAS2L”, “CBX”, and “CENP” were used as marker genes of G2M phase. The TUBB4B gene was removed from the default Seurat G2M marker genes because TUBB4B is also a marker of ciliated cells. The ratio of cycling cells of each culture method of a cell type was calculated as the average ratio of S to G2M phase cells of individuals.

### Integration of OSCA and in vivo tissue scRNA-seq data

To compare transcriptomes between in vitro human lung cells and in vivo human lung tissues, we integrated our in vitro single-cell datasets with in vivo tissue datasets of human lung cell atlases from the European Genome-phenome Archive (EGA) under accession number EGAS00001004344^1^. We used 1–50 PCs and SCT to transfer anchors between in vitro cell and tissue data using FindTransferAnchors^37^. After MapQuery was performed, using integrated PCs, we regenerated UMAP and Louvain clusters. Cell types were identified by annotation using scHCL (v0.1.1)^38^ and Azimuth^37^ and manual curation with known marker gene expressions.

### Cell type annotation prediction using CellTypist

The Python package CellTypist (v.1.2.0)^39^ was used to perform cell type annotation prediction with logistic regression models. All the models provided by CellTypist trained with lung single-cell data were used on our data for cell type annotation prediction and validation. We also used lung single-cell data and annotated metadata from P.K.L. Murphy et al. as a training dataset to create the CellTypist model. Default parameters were used for model building. All the predictions were made with majority voting.

### Rare cell score

For cell type identification, scHCL was used to match each cell to annotated scRNA-seq data^38^. Within 23 clusters determined by the Louvain clustering algorithm, Cluster 22 exclusively expressed canonical marker genes of rare airway epithelial cell populations, such as tuft cells, ionocytes and pulmonary neuroendocrine cells (PNECs), and was primarily labeled ‘Basal.cell.Airway. Epithelium_Plasschaert’ or ‘Ionocyte.Airway.Epithelium_Plasschaert’. We defined the scHCL_score of ‘Basal.cell.Airway. Epithelium_Plasschaert’ or ‘Ionocyte.Airway.Epithelium_Plasschaert’ as the rare cell score.

### Rare cell population lineage trajectory

Single-cell RNA-seq data across sample origins were reintegrated using Seurat’s CCA algorithm^37^. Monocle (v3.1.0.0) was then used to determine the paths that cells can take as they develop along the UMAP via the learnGraph function^40^. Cells were then ordered along the identified trajectory of pseudotime using the orderCell function assuming that the cluster predominantly comprised of basal cells served as the initial root group. Then, we used the prepPseudotimePlotDatasets function adopted from Goldfarbmuren et al. to view the expression of key marker genes along pseudotime on a shared scale^41^. This approach enabled the generation of smoothed expression curves across pseudotimes normalized between zero and one.

### Single-cell multiome data analysis

Single-cell multiome data were aligned to the GRCh38 reference genome using cellranger-arc (v2.0.0)^34,42^. Gene expression data from each single cell followed an identical pipeline, which was used in 3’ scRNA-seq data with Seurat (v4.0.3)^37^. The Signac (v1.6.0) package^43^ was used to filter, cluster, and visualize the assay for transposase-accessible chromatin (ATAC) data. Cells with more than 200 open peaks and peaks detected in more than 10 cells were used. Using NucleosomeSignal and TSSEnrichment, cells with less than 4 nucleosome_signal and more than 2 TSS.enrichment were normalized with RunTFIDF, FindTopFeatures, and RunSVD. To identify TAB cell-specific overrepresented motifs, we used FindMotifs to compare TAB cells and basal-2 cells. The most significantly overrepresented motif profiles were represented using the JASPAR^44^ database. Motif activity scores were calculated with chromVAR with bSgenome.Hsapiens.UCSC.hg38^45^.

### Identification of enriched genes, pathways, and immune activity

Normalized expression profiles for each culture method or subcluster were compared using the Wilcoxon rank sum test with the FindMarkers function^37^. Genes showing an adjusted *p* value < 0.05 were considered differentially expressed genes (DEGs). Gene set enrichment analysis (GSEA) was completed using GSEAPreranked with DEGs ranked by log_2_FC. Hallmark and C5: Biological process were tested for enrichment. Gene sets with FDR q-values < 0.01 were considered significant. scRNA-seq data from tissues of lung disease patients and organoids with cytokine treatment were reference mapped to OSCA integrated with tissues to predict cell types and subclusters of each cell. We used idiopathic pulmonary fibrosis (IPF), chronic obstructive pulmonary disease (COPD), cystic fibrosis (CF), healthy control, and fetal lung tissue data from GEO using GSE135893, GSE150674, and EMBL-EBI ArrayExpress databases (E-MTAB-8221), respectively^4,46,47^. Before reference mapping, each query data point was manipulated with SCTransform, RunPCA, and RunUMAP as with the reference OSCA data. After transfer anchors, the ratio of subclusters predicted by MapQuery was calculated by (number of immune-primed cells)/(number of all basal or ciliated cells) for each sample.

### Single-cell RNA-seq data analysis with virus-infected samples

We used a chimeric reference genome with GRCh38 and NC_045512.2^48^, NC_019843.3^49^, and NC_002016.1 ~ NC_002023.1^50^ for SARS-CoV-2, MERS-CoV, and influenza A, respectively. All processes, including alignment, normalization, and filtering, were the same as those in the pipeline used to construct OSCA. Infected cells are defined as cells expressing viral genes. IFN and entry scores were measured using AddModuleScore in the Seurat package with genes related to interferon signaling and viral entry factors, respectively^51^.

## Results

### Biobanking of human primary distal airway organoids

**Fig. 1:**
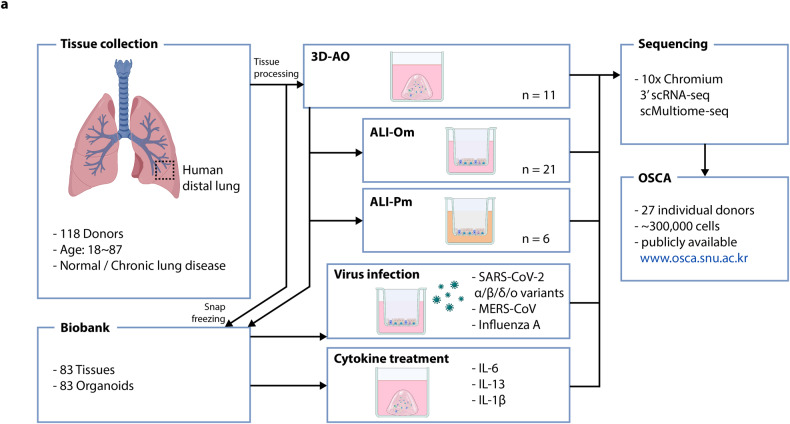
Establishing organoid biobanks and a single-cell atlas of human primary distal airway organoids. **a** Schematic workflow of organoid biobank generation, single-cell transcriptome analysis, and respiratory virus infection and cytokine treatment of organoids. 3D-AO (3D airway organoid), ALI-Om (air-liquid interface culture with organoid medium), ALI-Pm (air-liquid interface culture with PneumaCult medium), OSCA (Organoid Single-Cell Atlas). n number of sample profiling.

We established biobanks of human primary airway organoids with 83 cryopreserved organoid lines, matched snap-frozen tissues, and genotyping data. Surgically resected human distal lung tissues were enzymatically dissociated into single cells used to generate 3D airway organoids (Fig. 1)^17^. Established organoids were maintained by passaging up to 3 times (83/118; 70% success rate) and biobanked. DNA panel sequencing for genotyping was performed on matched frozen tissues.

To test the impact of culture conditions on cellular behaviors and gene expression programs in vitro, we directly compared the two most widely used in vitro human airway models, namely, 2D ALI and 3D organoid cultures. Airway organoids were dissociated into single cells to establish a submerged 2D ALI culture (Fig. 1a)^52^. We used the same medium for 3D airway organoid (referred to hereafter as 3D-AO) and 2D ALI culture (referred to hereafter as ALI-Om) to eliminate the medium as a variable (see Methods). We also grew the cells in a conventional ALI culture that results in mucociliary differentiation using commercially available PneumaCult medium (referred to hereafter as ALI-Pm) (Fig. 1a; see Methods)^53–55^. We used immunofluorescence (IF) staining for airway lineage markers to confirm the spatial distribution of differentiated cells within the pseudostratified epithelium of 3D-AOs and ALI-Oms, including basal, goblet, club, and ciliated cells (Supplementary Fig. 1a).

### Single-cell transcriptome atlas reveals preserved cellular diversity of human primary airway cells across in vitro multiculture systems

**Fig. 2:**
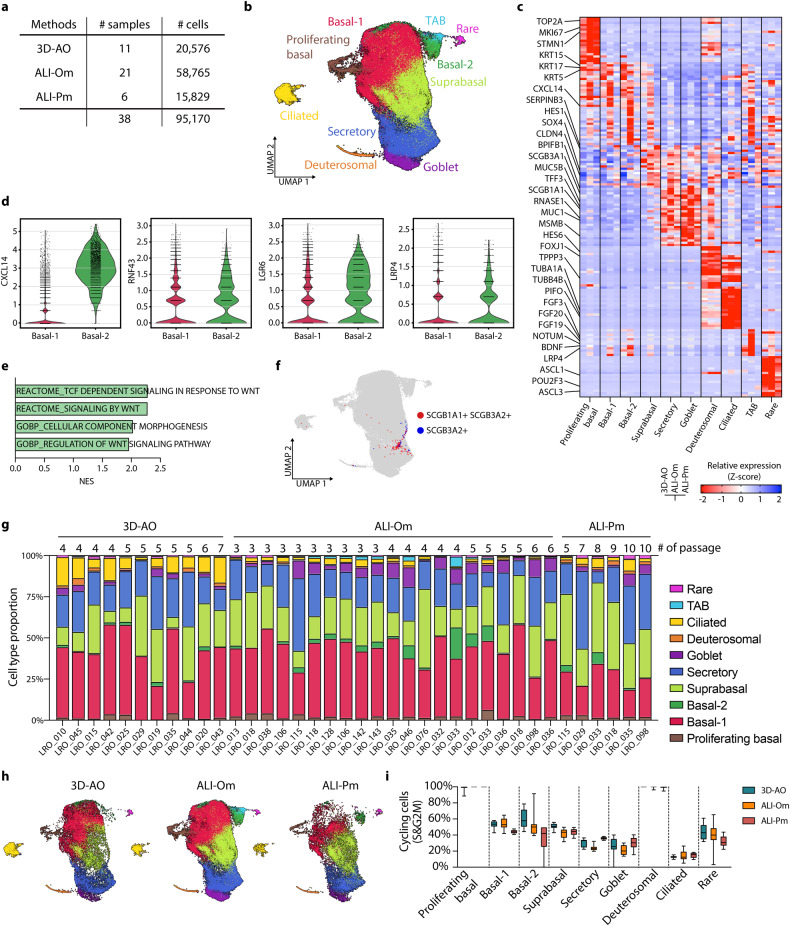
Single-cell transcriptomic profiling of in vitro human airway multiculture systems. **a** The number of profiled samples and cells for each culture method. **b** UMAP representing the scRNA-seq data of in vitro human primary airway cells indicated in **a**. Ten different airway epithelial cell types are visualized with colors. TAB, transitioning airway basal. **c** Heatmap showing differential marker gene expression for epithelial cell types. Each cell type column: 3D-AO (left), ALI-Om (middle), ALI-Pm (right). **d** Violin plot showing the expression of the indicated genes in basal-1 and basal-2 cells. **e** Bar plot showing the pathways enriched in basal-2 cells compared with basal-1 cells. Normalized enrichment score calculated by GSEA of DEGs. *P* < 0.01. **f** UMAP showing the expression of *SCGB1A1* and *SCGB3A2* in airway cells in vitro. **g** Bar chart depicting the proportion of various airway cell types in each of the 38 samples, which are ordered by culture method and passage number. **h** UMAP showing the distribution of each culture dataset from **b**. **i** The ratios of cycling cells for each culture condition, calculated as (number of S + G2M phase cells)/(number of all cells) for different cell types. Box plots with 10–90 percentile whiskers contain each sample derived from different donors.

We generated a single-cell transcriptional atlas of human primary distal airway organoids cultured under the three different conditions described above. We used the 10X Genomics platform to measure the single-cell transcriptome of 3D-AOs, ALI-Oms, and ALI-Pms derived from 27 individuals, including 19 normal and 8 diseased lungs (Fig. 2a and Supplementary Tables 1 and 4). A total of 95,170 cells passed quality control, and after raw data quality control, each of the separately constructed datasets was integrated via canonical correlation analysis (CCA) batch correction (see Methods). We annotated the major airway epithelial cell types using canonical cell type marker genes. The results showed that all 27 organoid lines contained all of the major cell types, regardless of culture method. The profiles of in vitro multiculture systems faithfully matched their in vivo counterparts: basal cells (*TP63, KRT5, KRT15*), secretory club cells (*SCGB1A1, BPIFB1*), goblet cells (*MUC1, MUC5B, MSMB*), and ciliated cells (*FOXJ1, PIFO, TPPP3, SNTN*) (Fig. 2b, c, g, h; Supplementary Fig. 1b; and Supplementary Fig. 2). We identified two subsets of basal cells (basal-1 and basal-2), with basal-2 cells expressing higher levels of *CXCL14, RNF43, LGR6*, and *LRP4* than basal-1 cells (Fig. 2b–e and Supplementary Fig. 1b). Notably, our large-scale single-cell profiling allowed us to detect SCGB3A2^+^ cells that were recently found in human lung terminal bronchioles (Fig. 2f)^2,3,56^. Given the active growth of organoids, we were able to capture cellular transitional states that are readily apparent in the homeostatic lung. For example, a group of organoid cells coexpresses basal and secretory cell marker genes (*KRT5*, *SCGB1A1*), suggesting suprabasal cells in the process of differentiating from basal to luminal secretory cell states. We also found a distinct cluster of deuterosomal cells that expressed both secretory and ciliated cell marker genes (*SCGB1A1*, *FOXJ1*), indicating cells in a transient state from secretory to ciliated cells. As expected, a subset of basal cells was actively proliferating (which we named proliferating basal cells), whereas differentiated cells demonstrated low cycling activity (Fig. 2c, i and Supplementary Fig. 1b). Interestingly, deuterosomal cells, which are precursors of ciliated cells, displayed remarkable cycling activity in both organoids and lung tissues (Fig. 2i and Supplementary Fig. 5c). Despite the differences in their proportions, all 27 in vitro lines maintained relatively comparable cellular diversity and states independent of culture conditions or passage numbers (Fig. 2g–i). There were also no discernable differences correlating with age, sex, smoking status, and disease background (Supplementary Fig. 1c–g). These datasets are available for interactive analysis at the Organoid Single-Cell Atlas (OSCA: http://osca.snu.ac.kr).

### Large-scale single-cell multiomics of in vitro multiculture models to track rare airway epithelial lineage differentiation

Recent single-cell transcriptome studies of human primary lung tissues allowed for the mapping of cellular composition and lineage hierarchy at the single-cell level^1–3,57^. However, epithelial cell types such as tuft cells, ionocytes, and pulmonary neuroendocrine cells (PNECs) are rare and therefore not amenable for either deep molecular characterization or inferring lineage trajectories from in vivo lung atlases^58^. Notably, our large-scale single-cell transcriptome profiling enabled us to detect all of these rare airway epithelial cell types in both 2D ALI and 3D organoid models: tuft cells (*POU2F3*), ionocytes (*FOXI1*), and PNECs (*ASCL1*) (Fig. 2b, c, g, and h and Supplementary Fig. 1b). To build a pipeline for tracking lineage trajectories of these rare epithelial cell types, we first tested the in vitro lineage relationship between basal cells and secretory cells, which is a well-established in vivo lineage hierarchy of basal to secretory cells in the lung^18^. Clusters of proliferating basal, basal-1, suprabasal and secretory cells were extracted and reintegrated from our single-cell datasets for further analysis. As expected, pseudotime trajectory analysis revealed the differentiation path of basal cells to secretory cells, ensuring the fidelity of in vitro differentiation programs compared with in vivo lungs (Supplementary Fig. 3a–d). Reclustering secretory, deuterosomal, and ciliated cells also showed the differentiation trajectories from secretory cells to ciliated cells via deuterosomal cells, as previously reported (Supplementary Fig. 3e–h).

**Fig. 3:**
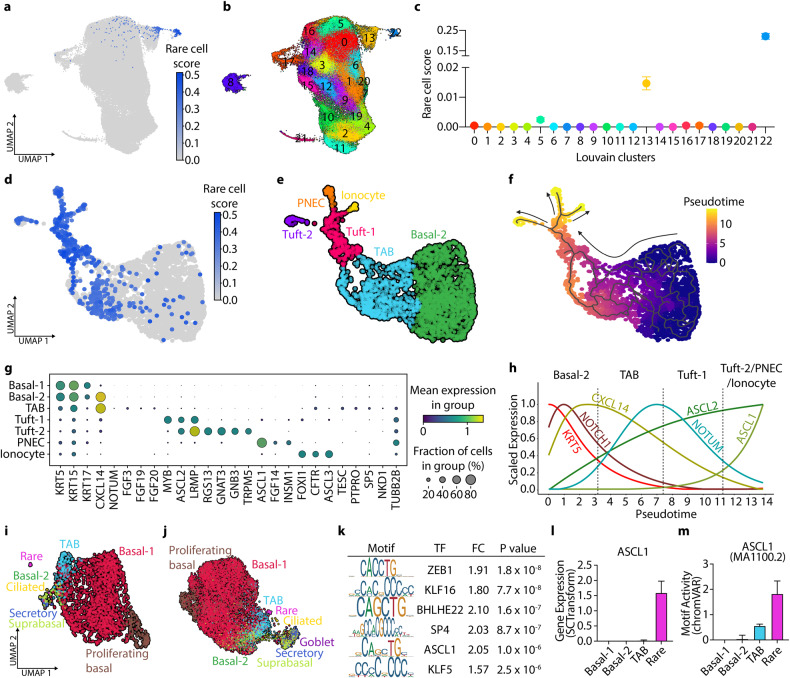
Combination of scRNA-seq and scATAC-seq to trace the lineage trajectory of rare epithelial cell types. **a** UMAP visualization of a rare cell score calculated from an enrichment score based on transcriptome similarity with a rare cell. **b** UMAP labeled with Louvain clusters, colored by 23 separate clusters. **c** Mean scatter plot with 95% CIs showing an average rare cell score of each Louvain cluster. **d** Reclustered UMAP with Louvain Clusters 13 and 22 in **b**, **c**, colored by a rare cell score (*n* = 2,962 cells). **e** Reclustered UMAP revealing all of the expected rare epithelial cell types, such as ionocytes, tufts, and pulmonary neuroendocrine cells (PNECs), and a new transitional airway basal (TAB) cell state. **f** Pseudotime trajectory analysis measured by Monocle3. **g** Dot plot representing differential marker gene expression for each cell type and state. **h** Smoothed expression curves across pseudotime starting from basal-2 (*t* = 0) show the relative expression change of the indicated genes. (**i,** **j**) UMAP with gene expression data **i** and ATAC peaks **j** obtained from 10× multiome sequencing. **k** Top six motif matrices and transcription factors calculated by logistic regression with basal-2 and TAB-specific open regions. (**l**, **m**) Bar plot with 95% CIs showing normalized expressions measured by SCTransform **l** and motif activity measured by ChromVar (m) for ASCL1.

We then investigated lineage differentiation of rare cell types by establishing an enrichment score based on transcriptome similarity between each cell and known reference rare cells (referred to hereafter as rare cell score; see Methods)^38^. Among 23 unbiased Louvain clusters, Cluster 22, which retained rare epithelial cell types, had the highest rare cell score in vitro airway cells (Fig. 3a–c). Interestingly, among nonrare lung epithelial cells, basal-2 cells (Cluster 13) had the highest rare cell score, leading us to hypothesize that basal-2 cells could be precursor cells for rare cell differentiation (Fig. 3a–c). We thus extracted and reclustered cells in Clusters 13 and 22 (Fig. 3d, e). Notably, basal cells with high rare cell scores were closely aligned with clusters of distinct rare cell populations. Furthermore, based on the expression level of basal cell marker genes (*KRT5*, *KRT15*), rare cell scores, and pseudotime trajectory analysis, we were able to dissect transitioning airway basal (TAB) cells, which showed lower expression of basal cell marker genes and were closely associated with basal-2 and rare cell types (Fig. 3e–h and Supplementary Fig. 4). TAB cells were specifically marked by the expression of *NOTUM*, *FGF3*, and *FGF19*, suggesting a distinct molecular program modulating TAB cells in this transition (Fig. 3g, h and Supplementary Fig. 4). To better understand the relationship between TAB cells and rare airway lineages, we applied 10x single-cell multiomics to 3D-AOs. Significantly, analysis of a single-cell assay for transposase accessible chromatin using sequencing (scATAC-seq) demonstrated that TAB cells annotated by gene expression profiles were also closely assigned to rare cell populations (Fig. 3i, j). We further determined transcription factor (TF) binding sites that were specifically opened in TAB cells (Fig. 3k). Interestingly, TF binding sites for *ASCL1*, a marker gene for PNECs, were opened in TAB cells, whereas its expression was restricted to PNECs, suggesting that TAB cells could represent primed cell states for differentiating into PNECs from basal cells (Fig. 3l, m). Additionally, tuft cells were further classified as tuft-1 (*ASCL2*) and tuft-2 (*TRPM5*) cells based on unique signatures (Fig. 3e, g and Supplementary Fig. 4)^59–61^.

### A distinct transcriptomic signature of the immune response in in vivo human lung tissues versus in vitro organoid models

**Fig. 4:**
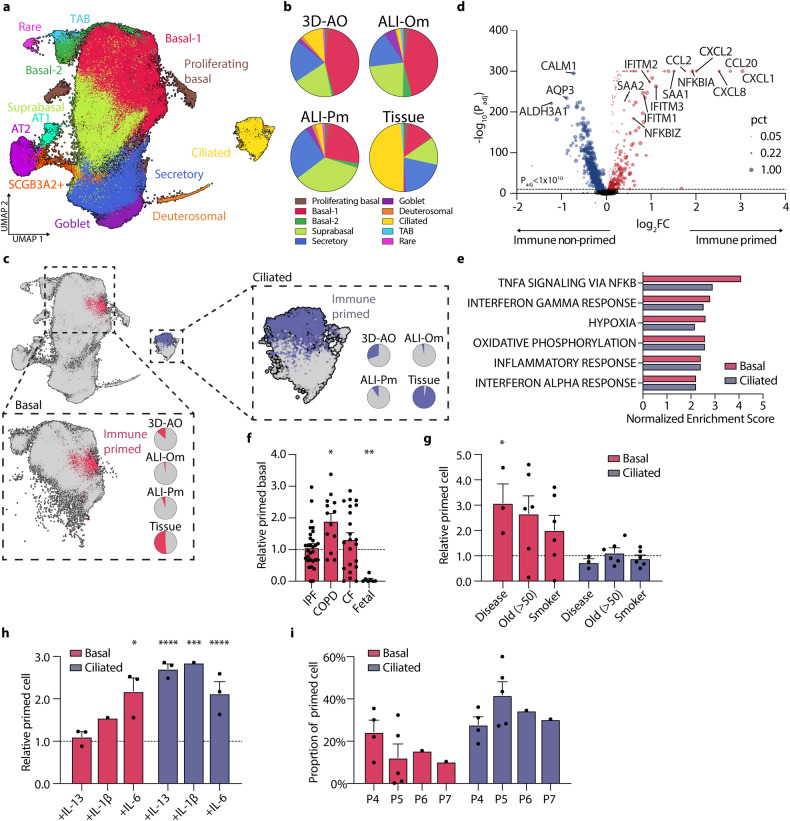
Comparative scRNA-seq analysis of in vitro airway models and in vivo lung tissues identifies immune-primed cell states. **a** Integrated UMAP visualization with cells in in vitro airway models and in vivo human lung tissues (*n* = 104,577 cells total)^1^. Note that alveolar lineage cells such as alveolar type 1 (AT1) and type 2 (AT2) cells are labeled only in in vivo lungs but not in in vitro cultures. **b** Pie charts showing the proportion of different cell types in each culture method and tissue. Only airway cells are used to measure the proportion of cell types in vivo lungs. **c** UMAP showing distinct immune-primed cell states only in basal and ciliated cells. Pie charts show the greater proportion of immune-primed basal and ciliated cells in in vivo lungs than in in vitro lung cells. **d** Volcano plot showing differentially expressed immune-responsive genes in immune-primed vs. nonprimed basal cells. Dot size represents the ratio of immune-primed basal cells (pct) expressing each gene. **e** Bar plot showing the pathways enriched in immune-primed basal and ciliated cells compared with nonprimed cells. Normalized enrichment score of the HALLMARK gene set using GSEA. *P* < 0.001. **f** Relative proportions of immune-primed basal cells in in vivo human lung single-cell transcriptome data with chronic respiratory disease^4,47,62^. Each value is normalized by their corresponding healthy controls. **g**, **h** Relative proportions of immune-primed basal and ciliated cells in 3D-AOs with donor characteristics **g** and cytokine treatments **h**. Each group was normalized to healthy **g** and vehicle-treated **h** samples. **i** The proportion of immune-primed basal and ciliated cells over multiple passages in 3D-AOs. *P < 0.05, **P < 0.01, ***P < 0.001, ****P < 0.0001, unpaired t test.

To determine how closely the cellular and molecular features of in vitro human airway cultures replicate those of in vivo human lung tissue, we integrated well-annotated data of epithelial cell clusters from the Human Lung Cell Atlas with our dataset (Fig. 4a and Supplementary Fig. 5a, b; see Methods)^1^. As previously stated, the cellular composition of in vitro models is comparable to that of lung tissues, whereas the proportions of each cell type differ (Fig. 4b and Supplementary Fig. 5c). In vitro airway cells contained more basal and intermediate cells, whereas epithelial cells from lung tissues had more differentiated cells. We then compared the molecular programs of in vitro airway cells with those of lung tissues. Significantly, gene set enrichment analysis (GSEA) of differentially expressed genes (DEGs) in cells derived from lung tissues and cultures revealed enriched gene expression patterns involved in immune responses in vivo lungs (Supplementary Fig. 5d). We attribute this result to the unique feature of in vitro airway models that, despite faithfully mimicking in vivo tissue characteristics, are entirely composed of epithelial cells and lack immune cells. Interestingly, only the subsets of basal and ciliated cells showed enriched expression of immune response genes (*CXCL1/2/8*, *CCL2/20*, *SAA1/2*, *IFITM1/2/3*) (Fig. 4c, d and Supplementary Fig. 5e, f). GSEA using hallmark gene sets of DEGs also showed that these subsets were characterized by increased expression of genes associated with immune and inflammatory responses, indicating ‘immune-primed cell states’ (Fig. 4c, e and Supplementary Fig. 5g, h). Interestingly, although basal and ciliated cells are molecularly and functionally distinct cell types, transcriptional differences in their respective immune-primed cells revealed strong similarities (Fig. 4e).

We next investigated the clinical relevance of immune-primed cells in chronic lung diseases. Using reference mapping, we combined our single-cell RNA sequencing (scRNA-seq) data with those from previous studies on patients with idiopathic pulmonary fibrosis (IPF), chronic obstructive pulmonary disease (COPD), and cystic fibrosis (CF) (see Methods)^47,62,63^. We found higher proportions of immune-primed subsets in the basal cells of diseased lungs compared to their normal controls, whereas fetal lung tissues barely exhibited immune-primed cell states (Fig. 4f)^4^. We then further analyzed immune-primed cells in our airway organoids derived from normal and chronic respiratory disease lung tissues (Supplementary Table 1; see Methods). Organoids derived from diseased lung tissues revealed a significant increase in immune-primed basal cells compared to organoids derived from normal lung tissues (Fig. 4g). Furthermore, organoids derived from lung tissues of aged ( > 50) or smoking donors also showed an increase in immune-primed basal cells compared to organoids derived from lung tissues of young or nonsmoking donors, respectively (Fig. 4g). However, we observed no discernable increase in immune-primed ciliated cells despite their absolute proportion being greater than that of basal cells regardless of donor characteristics. We next investigated whether inflammatory cytokines could be used to mimic the in vivo immune microenvironment in organoid cultures. Treatment with proinflammatory cytokines, such as IL-1β and IL-6, induced immune-primed basal and ciliated cells in 3D-AOs derived from healthy lungs, demonstrating that human primary airway organoids replicate in vivo immune responses by modulating microenvironmental culture conditions (Fig. 4h). Notably, ciliated cells were more responsive to inflammatory stimuli than basal cells. We also noticed a trend of a gradually decreasing proportion of immune-primed basal cells in 3D-AOs over multiple passages, indicating that cell states influenced by local microenvironmental signals may be lost during extended in vitro cultures (Fig. 4i).

### Transcriptomic responses of human airway organoids to respiratory virus infections

**Fig. 5:**
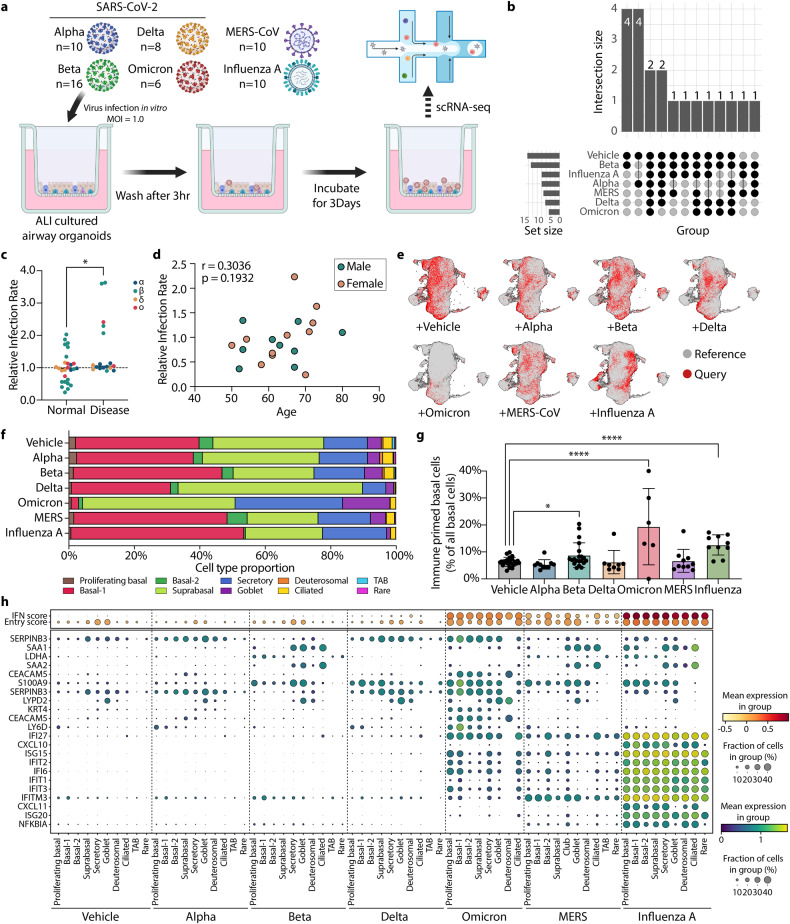
Transcriptomic responses of airway cells to respiratory virus infections. **a** Schematic workflow of virus infection and scRNA-seq of ALI-Oms. **b** UpSet plot showing intersections of sequenced cells in each infection group. **c** Relative infection rate of SARS-CoV-2 (Alpha, Beta, Delta, and Omicron) in ALI-Om cells derived from normal and diseased lung tissues. Values are normalized to the average infection rate of normal samples for each virus separately. *P < 0.05, unpaired t test. **d** Relative infection rate of SARS-CoV-2 viruses with age and sex of each infected sample. Two-tailed Pearson correlation. **e** UMAPs highlighted with query cells representing reference mapped cells from each infection group. Reference represents OSCA integrated into lung tissue data, and query represents each ALI-Om data point infected with the indicated viruses. **f** Stacked bar charts showing the proportion of cell types in each infection group. **g** Bar plot with 95% CIs representing the ratio of immune-primed basal cells in each infection group. *P < 0.05, ****P < 0.0001, unpaired t test between vehicle and each infection group. **h** Dot plot representing the expression levels and fraction of cells that express the indicated immune responsive marker genes according to the cell type and infection groups.

Finally, we utilized our platform to model various respiratory virus infections and compare transcriptomic signatures of infected human primary airway epithelia (Fig. 1a). Established ALI-Om cells derived from 20 individual tissue donors were used for infection with SARS-CoV-2 variants (B.1.1.7 Alpha, B.1.351 Beta, B.1.617.2 Delta, and B.1.1.529 Omicron), MERS-CoV, and influenza A viruses (Fig. 5a, b and Supplementary Tables 2 and 5). We first assessed the expression of the *ACE2* and *TMPRSS2* genes, which are necessary for SARS-CoV-2 viral entry, in ALI-Oms during air-liquid differentiation by quantitative PCR (q-PCR) analysis. The expression levels of *ACE2* and *TMPRSS2* were upregulated at 3 weeks after the induction of differentiation, when the virus was delivered (Supplementary Fig. 6a, b). Transmission electron microscopy (TEM) analysis showed widespread SARS-CoV-2 Alpha viral particles in ALI-Oms at 72 h post-infection (Supplementary Fig. 6c). IF staining for nucleocapsid protein (NP) also confirmed the efficient infection of SARS-CoV-2 alpha and influenza A viruses in these cells (Supplementary Fig. 6d).

We conducted scRNA-seq analysis of ALI-Oms that were infected with SARS-CoV-2 variants, MERS-CoV, and influenza A viruses. Utilizing aligned reads on the viral reference genome, we measured the infection rate. ALI-Oms derived from diseased lung tissues showed a significant increase in infection rate compared to those from normal tissues (Fig. 5c). However, the age and sex of lung tissue donors had little effect on the infection rate (Fig. 5d). We then used our single-cell transcriptome platform as a reference dataset of untreated controls for the single-cell data of infected samples (Fig. 5e). The proportion of cell types was barely influenced by virus infection, with the exception of Omicron, which reduced basal-1 cell populations (Fig. 5f). Importantly, ALI-Oms infected with SARS-CoV-2 (Beta), Omicron and influenza A revealed significant increases in immune-primed basal subsets (Fig. 5g). However, we found no significant effect of age, sex, or disease history of donor tissues on the immune-primed cell states induced by virus infection in ALI-Oms (Supplementary Fig. 6e–g). As previously reported, we also demonstrated enriched interferon and viral entry scores in our infected cells, particularly with SARS-CoV-2 Omicron, MERS-CoV, and influenza A viruses (Fig. 5h)^51^.

## Discussion

Given the potential of human primary organoids as a platform to study human biology, it is critical to determine whether and to what degree organoids mirror the cellular and molecular events of complex biological processes in human tissues in vivo. We generated a massive single-cell atlas of three representatives in vitro human airway models and provided a pipeline for comparing in vitro datasets to those of in vivo human lungs.

We demonstrate that despite differences in the proportions of cellular composition, the cellular diversity and transcriptional cell states are significantly maintained in all in vitro models using 27 lines and are comparable to those of in vivo human lungs. These data strongly suggest that none of the culture methods are inferior and that there may be potential to perform cross-comparisons. Importantly, these cellular diversities are maintained throughout multiple passages up to 10, suggesting the stability of in vitro human primary airway models. Our large-scale single-cell transcriptome profiling allowed us to detect a distinct subset of basal cells expressing enriched Wnt signaling pathway genes and closely connected to rare cell differentiation, suggesting their unique stem cell characteristics, which requires further validation. Notably, we also captured a small subset of secretory cells expressing *SCGB3A2*, which were recently identified as potential progenitor cells differentiating from/to alveolar lineages in human distal terminal bronchioles^2,3^. Furthermore, despite decreasing cell cycle activity with differentiation trajectory, deuterosomal cells, precursors of ciliated cells, displayed high levels of cell cycling on an equal level with proliferating basal cells even in vivo lungs, suggesting their active contribution to the maintenance of ciliated cells in homeostatic lungs in vivo.

Recent studies have highlighted the emergence of intermediate cell states during lung regeneration and their clinical relevance in human lung diseases, including pulmonary fibrosis and lung cancer^64–66^. Given the features of in vitro models that recapitulate active regenerative contexts rather than homeostatic conditions, our single-cell datasets readily detected cell states that are closely associated with two different lineages and have the molecular characteristics of transitioning cell states. Significantly, our massive single-cell profiling of in vitro multiculture models allowed us to capture the differentiation dynamics of rare epithelial cell types, such as PNECs, ionocytes, and tuft cells, from basal cells, as previously reported in scRNA-seq of primary airway tissues^58,67^. We also detected subsets of tuft cells (tuft-1 and tuft-2) in our in vitro models, indicating the power of our platform to replicate lineage relationships of human lung tissues in vivo. A distinct basal subset of TAB cells was identified with the molecular characteristics of intermediate cell states between basal and rare cell types. Interestingly, the Wnt signaling pathway differentiates subsets of basal cells (Fig. 2d, e). The temporal increase in NOTUM expression, a negative regulator of Wnt signaling, in TAB cells suggests the dynamic regulation of Wnt activity during the differentiation of basal-2 cells to rare cell lineages via TAB cells (Fig. 3g, h). A recent study demonstrated direct differentiation of human airway basal cells to PNECs in a hypoxic context^68^. Notably, we found that the TF binding site for the PNEC marker gene ASCL1 is opened in TAB cells, whereas its expression is restricted to PNECs. This finding suggests the differentiation path of basal cells to PNECs via TAB cells. It will be interesting to investigate further whether TAB cells are specific cell states that transition to PNECs or whether they can also generate other rare cell types.

Because the lung epithelium is constantly exposed to environmental challenges in vivo, immune cells play an important role. Current in vitro models lack the ability to fully convey such dynamics, as demonstrated by our datasets. We identified unique immune-primed subsets of basal and ciliated cells enriched in human lung tissues relative to in vitro airway cultures. Notably, ciliated cells in vivo lungs are largely composed of an immune-primed cell population; as previously suggested, ciliated cells are readily responsive to inflammatory signals^19^. Interestingly, we also observed greater immune-primed cell states in 3D-AOs than in ALI-Oms and ALI-Pms, suggesting the potential contribution of extracellular matrix dimensionality to maintaining immune-responsive cells in vitro. Further analysis of scRNA-seq datasets from the lungs of chronic respiratory disease patients revealed that they contained more immune-primed subsets than healthy lungs. Notably, we discovered that smoking and lung disease histories, as well as the age of tissue donors, affected the status of immune-primed cells in established organoids, implying that cellular features of primary lung tissues are reflected in in vitro models. We did, however, observe a reduction in these features in organoids over multiple passages, which can be induced by the addition of proinflammatory cytokines. These findings demonstrate that cellular states can be shaped by microenvironmental factors, which impact their functional behaviors in vitro models. Thus, organoids may prove useful for understanding epithelial responses to resolution of active pulmonary inflammation and/or facilitate screening of compounds to augment immune responsiveness. Our findings provide important aspects of in vitro models as a platform for studying cellular characteristics and modeling lung diseases.

Given the urgent need to elucidate the pathogenesis of SARS-CoV-2 infection in the current pandemic, in vitro organoids have been extensively used to study virus tropisms and infection responses. We utilized our single-cell platform to compare the transcriptomic responses of human primary airway epithelial cells to various respiratory virus infections in vitro. Age, sex, and history of lung disease of tissue donors had little effect on the infection rate of different viruses in cultures. Furthermore, regardless of the donor characteristics, we discovered a slight increase in immune-primed subsets in infected cells. It would be intriguing to investigate whether additional inflammatory stimuli, such as adding immune cells or cytokines, trigger the expansion or activation of these primed cells in infected cells. It could also be useful to explore the differential responses of infected ALI-Oms derived from aged or/and diseased tissues to these stimuli. As previously reported, we also observed higher interferon responses in infected cells^69^. Most notably, respiratory viruses known to cause milder symptoms exhibited more pronounced changes in transcriptomic responses, including interferon-related signals. A recent study reported a higher innate interferon response in COVID-19 pediatric patients, which may correlate with reduced viral replication and milder disease progression in this population^51^. Our findings also demonstrated higher interferon signaling in ALI-Oms infected with SARS-CoV-2 Omicron variant and influenza A viruses, correlating with the milder symptoms of these pathogens.

In summary, by combining single-cell multiomics and in vitro human primary organoids derived from multiple individuals, we produced a powerful database that can be used as a platform to better understand human lung stem cell activities and lineage trajectories. Our approach can also serve as a preclinical model that guides optimized individualized treatments based on molecular profiling to avoid delays in the use of the most effective therapeutic regimens for existing and novel viruses.

## Supplementary information

Supplementary Information

Supplementary Table 1

Supplementary Table 2

Supplementary Table 3

Supplementary Table 4

Supplementary Table 5

## Data Availability

All data needed to evaluate the conclusions are present in the paper and/or the Supplementary Materials. All raw and processed sequencing data will be publicly available at Gene Expression Omnibus (GEO). Data can be visualized at http:// www.osca.snu.ac.kr.
