# Supplementary material for: A single-cell atlas of in vitro multiculture systems uncovers the in vivo lineage trajectory and cell state in the human lung
Source: Exp Mol Med. 2023 Aug 15;55(8):1831–42. doi: 10.1038/s12276-023-01076-z (PMC10474282; doi:10.1038/s12276-023-01076-z)
Supplement: Supplementary file 1 — Supplementary Information [file 12276_2023_1076_MOESM1_ESM.pdf]

This PDF file includes:

Supplementary Fig. 1 to 6
Legends for Supplementary Table 1 to 5

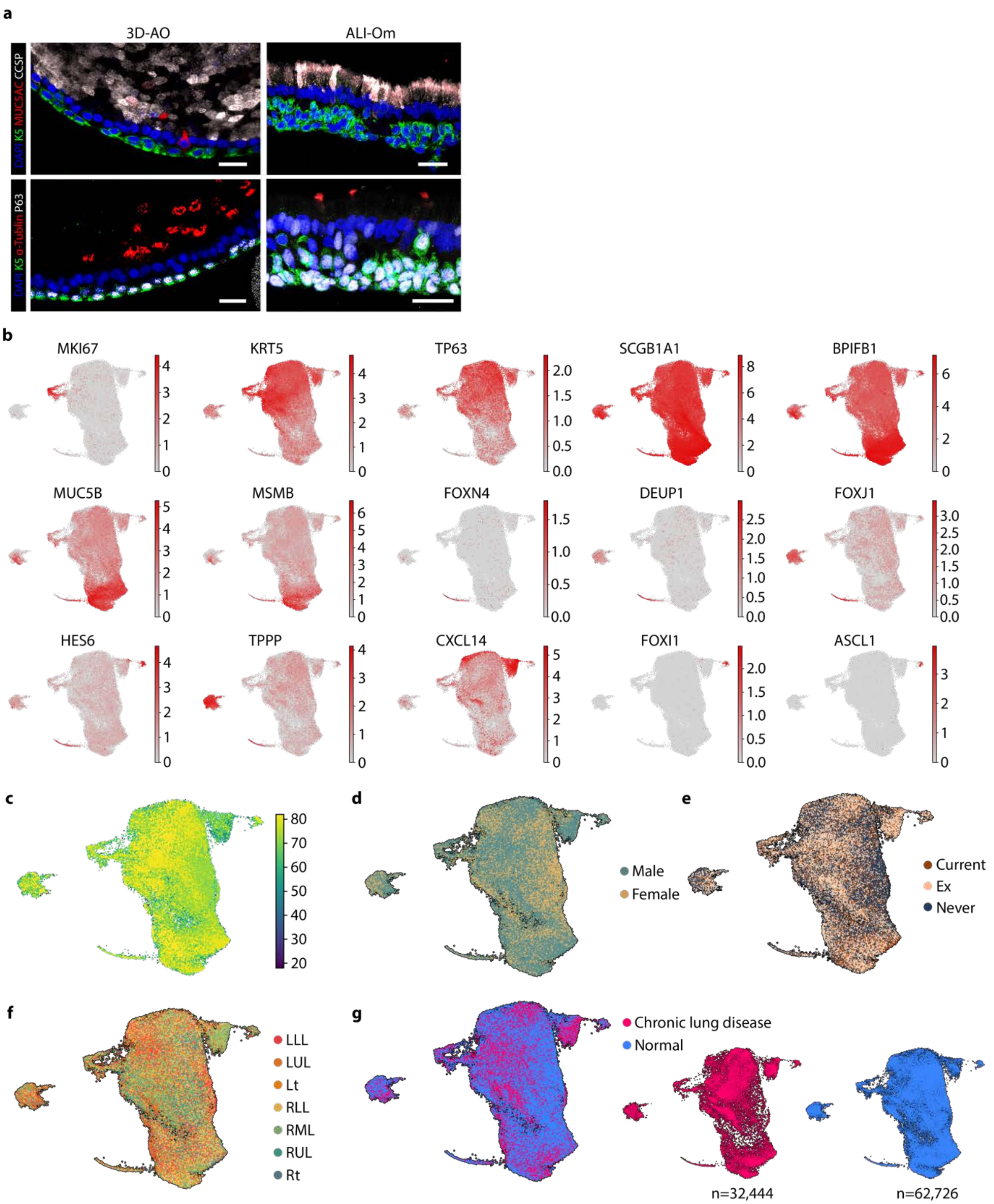

Supplementary Fig. 1. Direct comparison of *in vitro* human distal airway multi-culture models in the single cell resolution. (**a**) Immunofluorescent staining for airway lineage markers in 3D-AO and ALI-Om. nuclei (DAPI), basal cell (KERATIN-5, TP63), goblet cell (MUC5AC), club cell (CCSP), ciliated cell (ACETYLATED-TUBLIN). Scale bars, 30 μm. (**b**) Feature plot representing the expression of cell type marker genes. (**c** to **g**) UMAP labeled by age (c), sex (d), smoking status (e), specimen location (f) and comorbidity (g).

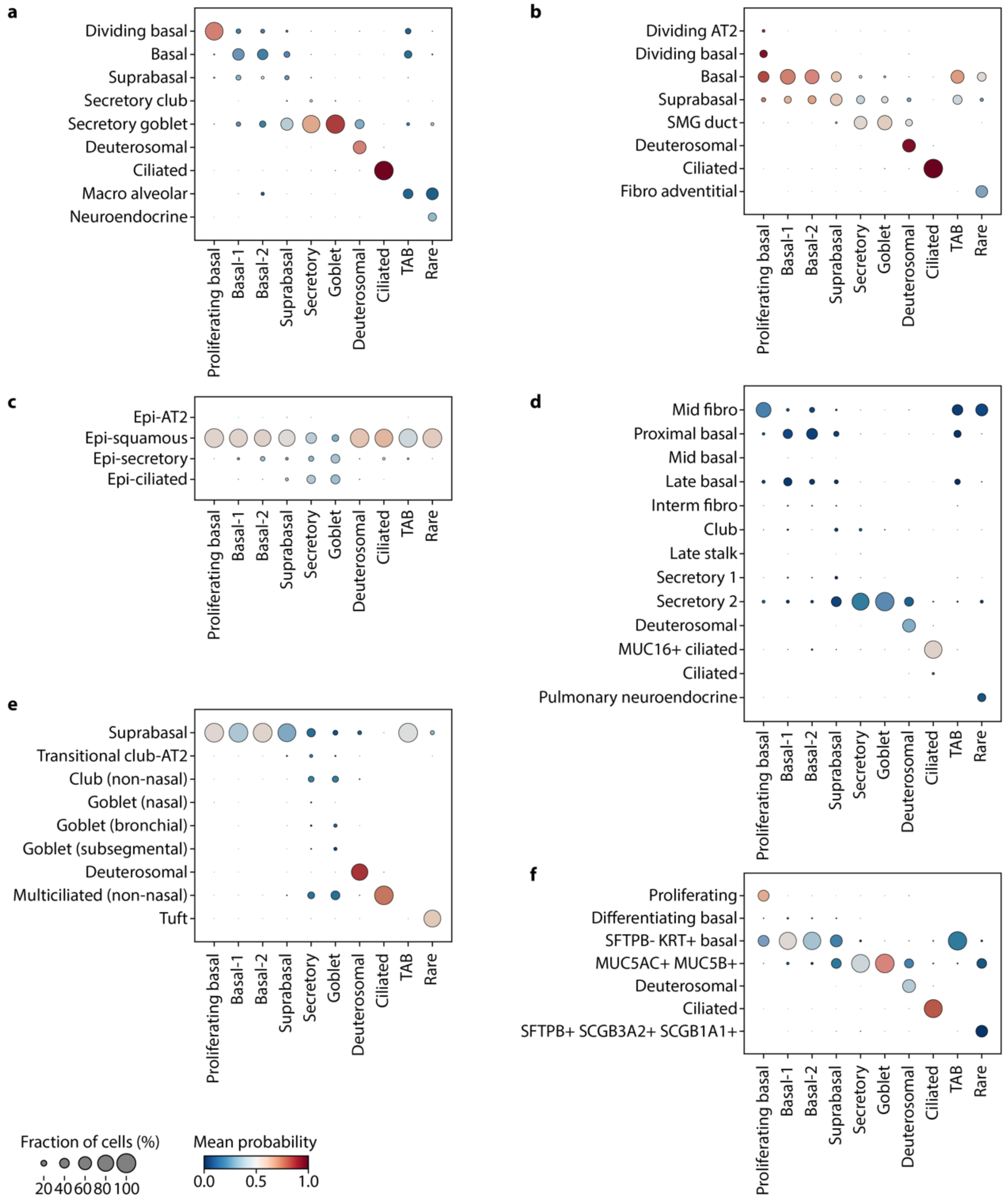

Supplementary Fig. 2. CellTypist cell type comparison with OSCA and known references. (**a** to **f**) Correlation between OSCA celltype and annotated celltype of other data calculated by CellTypist. OSCA celltype on row and query cell type on columns. E.Madissoon et al. (*69*) (Single Cell) (a), E.Madissoon et al. (*69*) (Single Nuclei) (b), X.Ren et al. (*70*) (c), P.He et al. (*71*) (d), L.Sikkema et al. (*72*) (e), P.K.L Murthy et al. (*2*) (f).

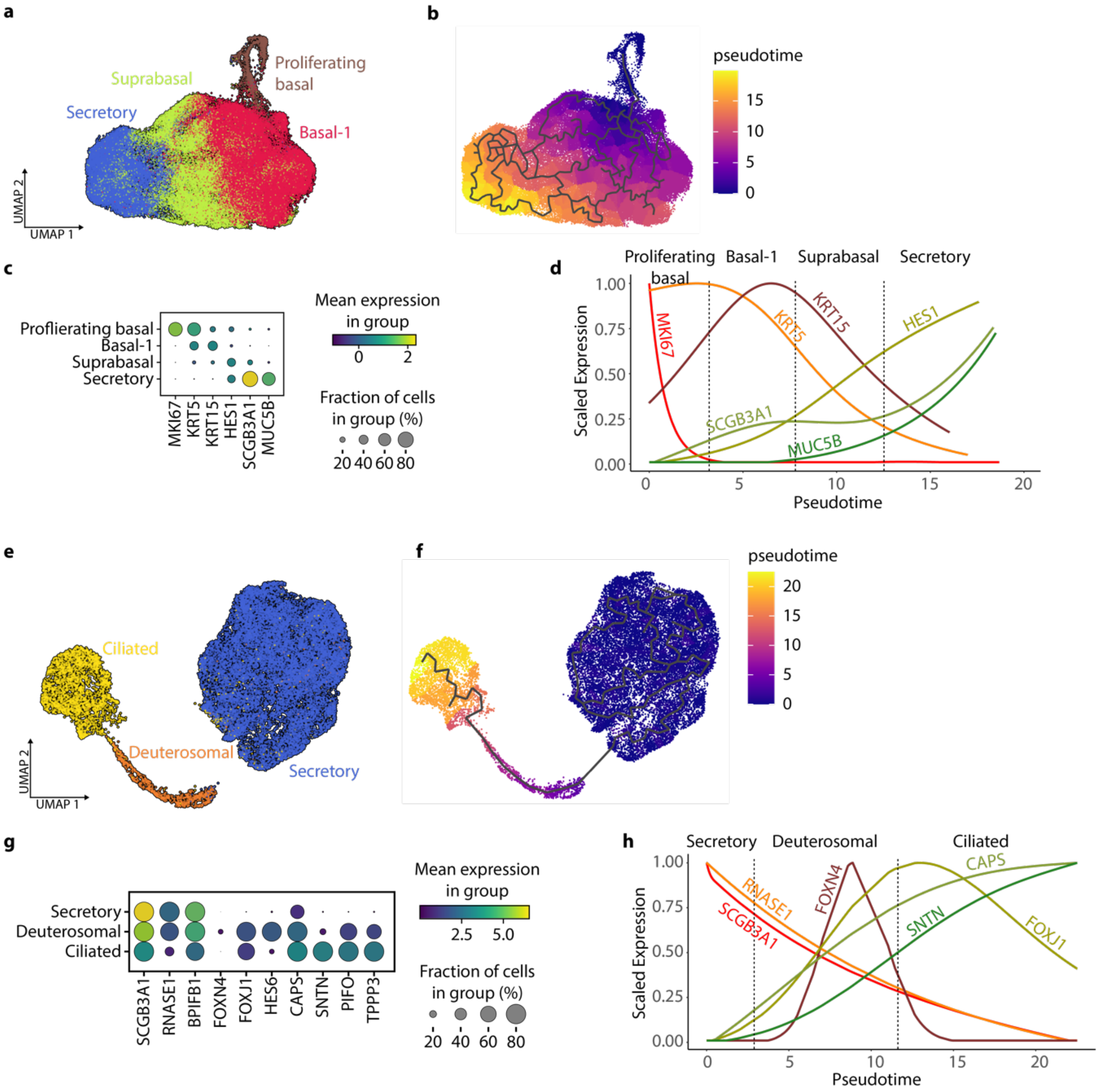

Supplementary Fig. 3. Transcriptomic features of *in vitro* human airway epithelial cell differentiation trajectories through pseudotime. (**a**) Re-clustered UMAP with proliferating basal, basal-1, suprabasal, and secretory cells in Fig. 2a. (**b**) Pseudotime trajectory analysis measured by Monocle3. (**c**) Dot plot representing differential marker gene expressions for indicated cell type. (**d**) Smoothed expression curves across pseudotime started from proliferating basal cells (t=0) show relative expression change of indicated genes. (**e**) Re-clustered UMAP with secretory, deuterosomal, and ciliated cells in Fig. 2A. (**f**) Pseudotime trajectory analysis measured by Monocle3. (**g**) Dot plot representing differential marker gene expressions for indicated cell type. (**h**) Smoothed expression curves across pseudotime started from secretory cells (t=0) show relative expression change of indicated genes.

a

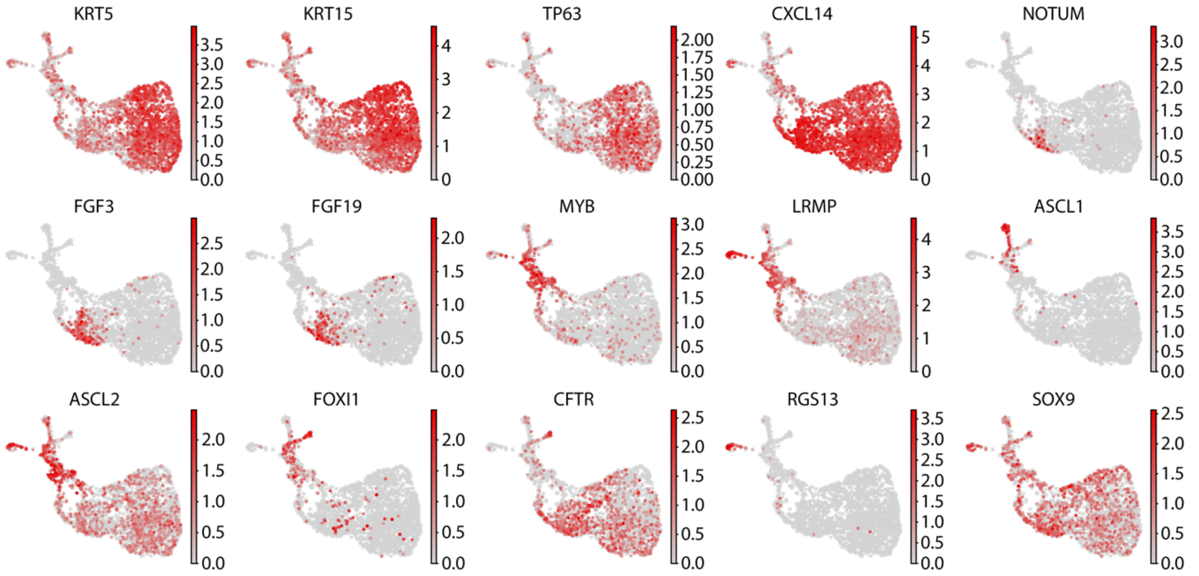

Supplementary Fig. 4. The expression of rare airway cell marker genes. (**a**) Feature plots representing the expression level of cell type marker genes.

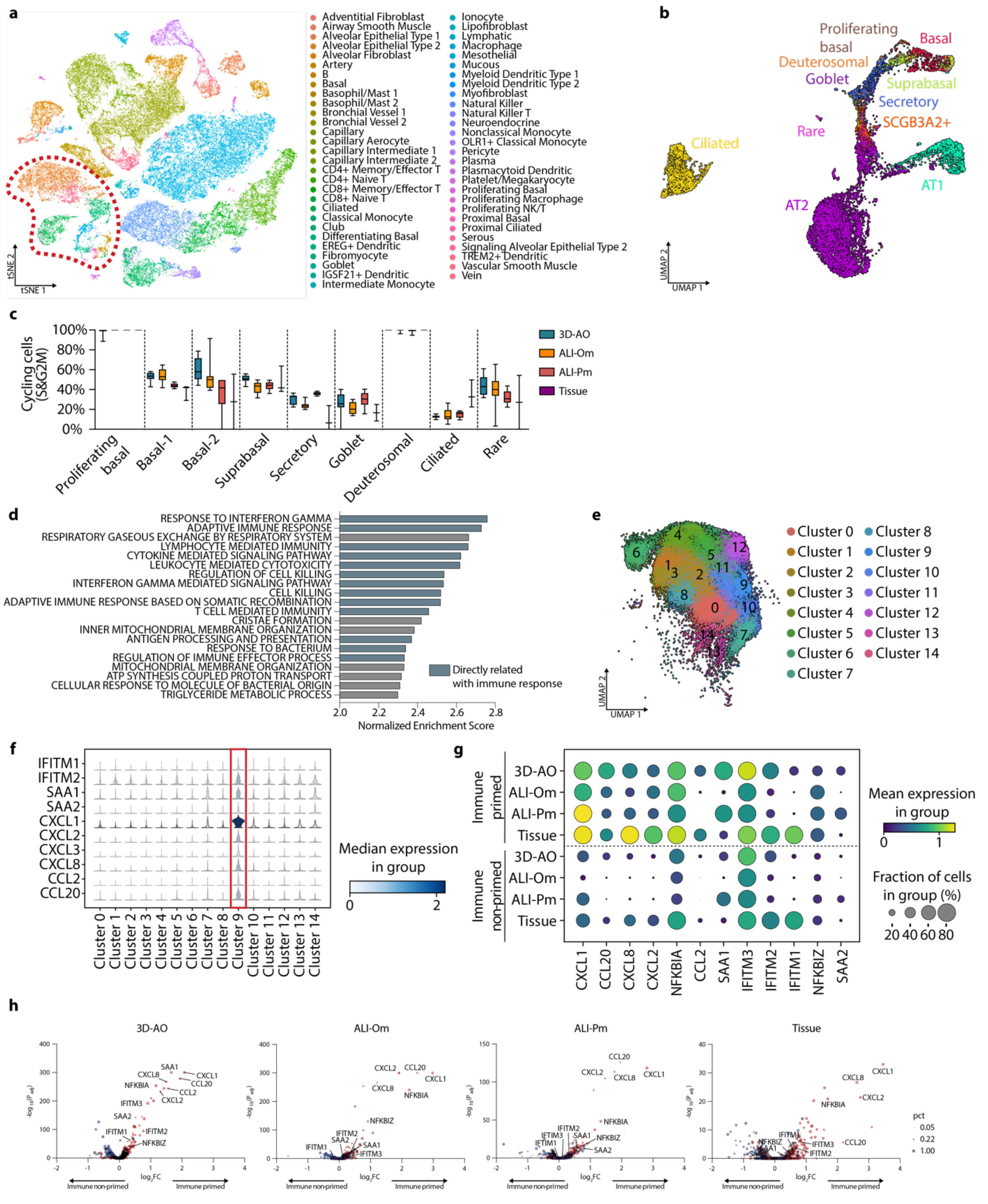

Supplementary Fig. 5. Comparison of *in vitro* and *in vivo* airway epithelial transcriptomes identifies a distinct immune primed cell state in human distal lungs. (**a**) tSNE plot of all human lung cell atlas data (*1*). Dashed line represents lung epithelial cells. (n = 65,662 total cells and

9,407 epithelial cells). (**b**) Re-clustered UMAP with lung epithelial cells in (a). (**c**) The ratios of cycling cells in three different *in vitro* cultures and *in vivo* lungs, calculated by (# of S + G2M phase cells)/(# of all cells) of different cell types. Box plots with 10-90 percentile whiskers contain each sample derived from different donor. (**d**) Bar plot showing the pathways enriched in *in vivo* lungs than *in vitro* airway models, ordered by normalized enrichment score calculated by GSEA of DEGs. Biological processes are activated in *in vivo* lungs. (**e**) UMAP visualization of basal cells in Fig. 4c, colored by 14 Louvain clusters. (**f**) Violin plots representing the higher expression levels of immune responsive genes in cluster 9 in (e). (**g**) Dot plot representing highly expressed genes in immune primed basal cells. (**h**) Volcano plot showing differentially expressed immune responsive genes in immune primed vs non-primed basal cells separated by culture methods. Up and down regulated genes in Fig. 4d are colored with red and blue.

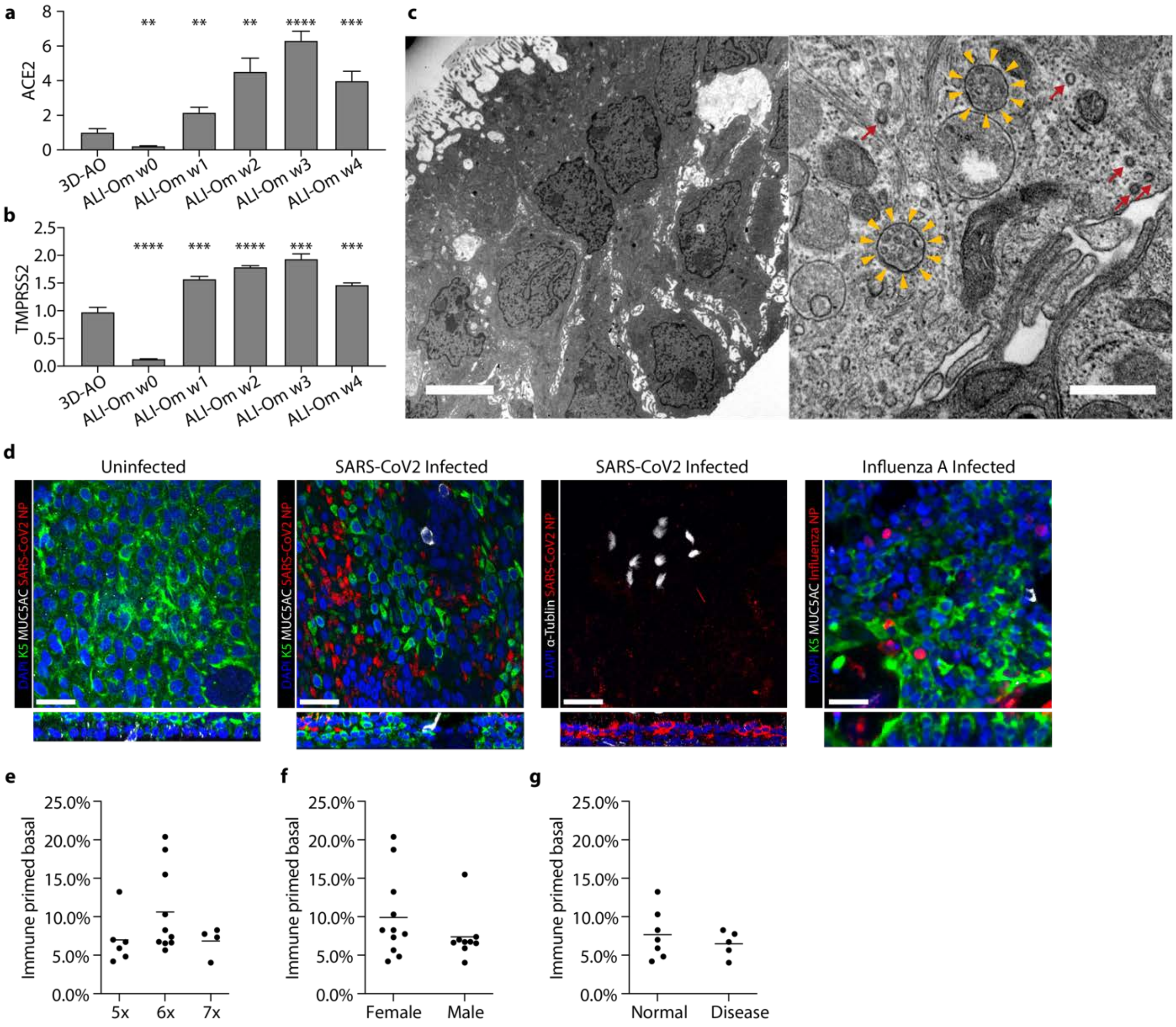

Supplementary Fig. 6. Respiratory virus infections in *in vitro* human primary airway models. (**a** and **b**) Relative expression levels (2^{-ddCt}) measured by quantitative PCR of major viral entry genes. **P < 0.01, ***P < 0.001, ****P < 0.0001, unpaired t test between 3D and each sample. (**c**) Transmission electron microscopy (TEM) image of SARS-CoV-2 alpha infection in ALI-Om. Red arrows indicate virus particles and yellow arrows mark virus vesicles. Scale bars, 5 µm (left), and 500 nm (right). (**d**) IF images of uninfected (left), SARS-CoV-2 infected (middle), and Influenza A infected (right) ALI-Oms. Scale bars, 30um. (**e** to **g**) The proportion of immune primed basal cells grouped by age (e), sex (f), and comorbidity (g).

Supplementary Tables

Supplementary Table 1. Organoid Sample ID and Sample Donor Clinical Metadata
Sample_Donor_Info: Details of Donor Age, Sex, Smoking Status, Comorbidity, Speciman Location, and Diagnosis for each donor.

Supplementary Table 2. Virus Infection
Virus_Strain_Info: Details of Virus Strains used for Lung Organoid Infection

Supplementary Table 3: Primer sequences for Quantitative RT-PCR
Sequence_Info: Primer sequences

Supplementary Table 4: scRNA-seq samples by culture method
Culture_Method: Information of Culture Methods used for each samples as shown in Figure 2. Colored tiles report the culture method used and single-cell RNA sequenced. The number of sequencing batches are reported as white numerals.

Supplementary Table 5: scRNA-seq samples by viral infection
Sample_Infection_Info: Details of Virus Infection Experimental Sets. Colored tiles report the infection experiments performed and single-cell RNA sequenced. The number of sequencing batches are reported as white numerals.
